# Berberine and its derivatives: mechanisms of action in myocardial vascular endothelial injury - a review

**DOI:** 10.3389/fphar.2025.1543697

**Published:** 2025-03-04

**Authors:** Wenhui Zhang, Siyi Guo, Jinjin Dou, Xiwu Zhang, Fan Shi, Chun Zhang, Huxiao Zhang, Xiaodong Lan, Yi Su

**Affiliations:** ^1^ Graduate School, Heilongjiang University of Traditional Chinese Medicine, Harbin, China; ^2^ First Clinical Medical School, Shandong University of Traditional Chinese Medicine, Jinan, China; ^3^ Department of Cardiovascular, The Fourth Hospital of Heilongjiang University of Traditional Chinese Medicine, Harbin, China; ^4^ Experimental Training Centre, Heilongjiang University of Traditional Chinese Medicine, Harbin, China

**Keywords:** berberine, myocardial vascular endothelial injury, mechanism of action, cardiovascular diseases, cardioprotection

## Abstract

Myocardial vascular endothelial injury serves as a crucial inducer of cardiovascular diseases. Mechanisms such as endoplasmic reticulum stress, apoptosis, inflammation, oxidative stress, autophagy, platelet dysfunction, and gut microbiota imbalance are intimately linked to this condition. Berberine and its derivatives have demonstrated potential in modulating these mechanisms. This article reviews the pathogenesis of endothelial injury in myocardial vessels, the pharmacological effects of berberine and its derivatives, particularly their interactions with targets implicated in vascular endothelial injury. Furthermore, it discusses clinical applications, methods to enhance bioavailability, and toxicity concerns, aiming to lay a foundation for the development of BBR as a therapeutic agent for cardiovascular diseases.

## 1 Introduction

Abnormal cardiovascular structure and function are inevitable consequences of aging. As the aging population continues to grow, the incidence of cardiovascular diseases (CVD) is also on the rise. Currently, CVD has surpassed cancer and other diseases as the leading cause of death worldwide (World Health Organization, 2024). Myocardial vascular endothelial injury (MVEI) is the primary prerequisite and foundational factor for the development of CVD. MVEI leads to the progression of atherosclerosis, acute coronary syndrome, coronary heart disease, heart failure, hypertension, and other CVD, as well as endocrine and hematological disorders (Mensah et al., 2019). Multiple manifestations, including reduced endothelial-dependent dilation, abnormal shear and tensile stress in blood vessels, and changes in plasma components, are early signs of MVEI, which can precede the onset of cardiovascular diseases. Therefore, MVEI is a powerful independent predictor of the occurrence and progression of CVD (Battson et al., 2017a).

Huanglian, a traditional Chinese medicinal herb widely used in clinical practice, is derived from the dried rhizomes of Coptis chinensis Franch., Coptis deltoidea C.Y. Cheng et Hsiao, or Coptis teeta Wall., all belonging to the Ranunculaceae family. It is primarily produced in the Sichuan region of China.Modern pharmacological research has shown that the chemical constituents of Huanglian, including isoquinoline alkaloids, lignans, flavonoids, and acidic compounds, contribute to its broad pharmacological activities, with isoquinoline alkaloids being the primary active constituents. Berberine (BBR), an isoquinoline alkaloid isolated from Huanglian, has the chemical formula C_20_H_18_NO_4_ and a molecular weight of 336.36. It appears as yellow needle-like crystals. Its hydrochloride salt is readily soluble in water, while the free base form is slightly soluble in water and almost insoluble in acetone, chloroform, or benzene. Derivatives of BBR, such as tetrahydroberberine (THBER), epiberberine (EPI), and berberrubine (BBB), are synthesized based on BBR’s structure. Pharmacological studies indicate that BBR and its derivatives play important roles in neuroprotection, stress mitigation, oxidative stress inhibition, anti-inflammatory actions, and metabolic regulation. Notably, extensive research has been conducted on the cardiovascular effects of BBR and its derivatives (Ren et al., 2021).

Traditional methods of administering BBR through oral and injectable routes have proven effective. However, BBR is characterized by low permeability, resulting in poor bioavailability. It may also be associated with a first-pass effect, leading to significant variability in drug concentration among individuals (Liu et al., 2016).Given the limitations of traditional administration methods, new drug delivery approaches have been developed in recent years, such as novel delivery systems, chemical modifications, microparticle delivery systems, and bioadhesive delivery systems. These strategies effectively enhance the release of active drug components and improve the binding rate of drugs to their targets. The use of intestinal absorption enhancers, such as penetration enhancers, bilosomes and their combinations, HP-β-CD-PEI polymers, and α-cyclodextrin, has also increased the bioavailability of oral drugs to some extent. Alternatively, pharmaceutical methods can be modified to reduce the hydrolytic degradation of drugs by intestinal microbiota or enzymes (Sultan et al., 2023; Zhang et al., 2017; Li et al., 2018). Drug modifications (such as PEGylation, poly amino acid modification, glycosylation, and molecular biology-based strategies) and drug delivery systems (such as polymer-based systems, lipid-based systems, inorganic nanoparticles for protein sustained-release delivery, and self-emulsifying drug delivery systems) have garnered widespread interest in biomedical applications (Nguyen et al., 2023; Zhu et al., 2020).Unfortunately, despite these new methods of modifying drug structures demonstrating improved biological properties, corresponding research on traditional plant-based drug extracts remains relatively limited, requiring more detailed experimental validation in the future.This article reviews recent studies on the pharmacological effects and signaling pathways of BBR and its derivatives in protecting against MVEI. Through a comprehensive review of literature from international databases such as Google Scholar and PubMed, the article aims to provide references for further in-depth research and clinical utilization of these compounds in CVD treatment.

## 2 Mechanisms of berberine and its derivatives in repairing myocardial vascular endothelial injury

### 2.1 Regulation of endoplasmic reticulum stress

The endoplasmic reticulum (ER) is a multifunctional organelle involved in the folding and translocation of secretory proteins and transmembrane proteins, regulating cellular Ca^2^⁺uptake, storage, and signaling, and mediating the production of cellular lipids, such as cholesterol, glycerophospholipids, and ceramides. When abnormal conditions occur in the ER, such as protein misfolding, accumulation of unfolded proteins, or calcium homeostasis imbalance, the ER responds by activating the unfolded protein response (UPR), the ER overload response, and apoptosis, thereby inducing ER stress (ERS) to protect and maintain ER stability (Ren et al., 2021; Perrotta, 2020). A study by Wang X. et al. (2021) demonstrated that ER stress-mediated apoptosis plays an important role in atherosclerosis and MVEI.

UPR is initiated by three transmembrane proteins: inositol-requiring enzyme 1 (IRE1), protein kinase-like endoplasmic reticulum kinase (PERK), and activating transcription factor 6 (ATF6). These transmembrane proteins regulate ERS by mediating protein degradation and inducing caspase-dependent apoptosis, as well as promoting the secretion of pro-inflammatory mediators through nuclear transcription factors, which lead to vascular inflammation and endothelial injury (Pérez-Torres et al., 2020; Battson et al., 2017b).Excessive or prolonged UPR causes severe damage to the ER function and triggers apoptosis via pathways such as the activation of C/EBP homologous protein (CHOP), c-Jun N-terminal kinase (JNK) signaling, or caspase-12 (Sozen et al., 2015).

**FIGURE 1:**
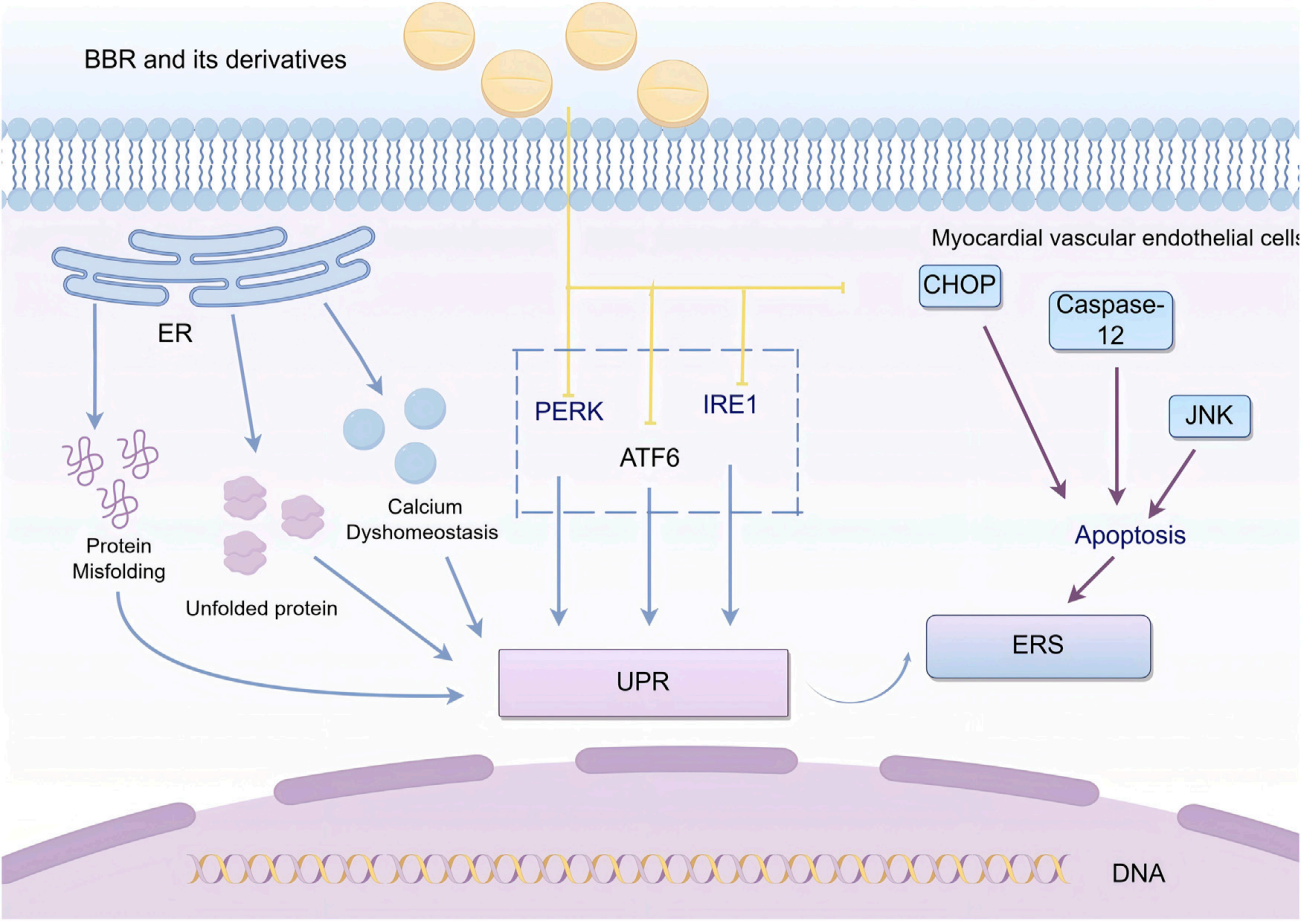
BBR and its derivatives are involved in endoplasmic reticulum stress mechanisms. They influence the endoplasmic reticulum stress by acting on the PERK, ATF6, IRE1, and CHOP pathways; sharp arrows (→) indicate stimulation, and blunt arrows (⊥) indicate inhibition. The figure was drawn using Figdraw.

Wang Y. et al. (2020) used a non-alcoholic fatty liver disease (NAFLD) mouse model to show that BBR inhibited the palmitic acid (PA) and lipopolysaccharide (LPS)-induced UPR in macrophages, significantly reducing the expression of CHOP mRNA. Wu Y. et al. (2021) found that BBR improved ERS in APP/PS1 transgenic mice by reducing the activity of glycogen synthase kinase 3β(GSK-3β) and inhibiting the activation of PERK. Li et al. (2022) demonstrated that BBR exerted vascular protection by inhibiting ERS in a vascular calcification rat model. Zhang et al. discovered that BBR could counteract ER stress-induced lipogenesis and mitigate hepatic steatosis, thereby preventing the progression to steatohepatitis and fibrosis by inhibiting the ATF6 pathway (Zhang et al., 2016).Furthermore, Xu et al. (2020) induced endothelial dysfunction in human coronary artery endothelial cells (HCAECs) by incubating them with serum from Kawasaki disease patients and then treated them with BBR for 24 h. They found that BBR suppressed the expression of PERK and IRE1, which were induced by Kawasaki disease serum, thereby repairing endothelial cell damage. Xu et al. (2020) also assessed ERS-related proteins such as HSP90B1 and DNAJC3, as well as upstream proteins such as P4HB, HSPA5, and VCP in HCAECs. They found that BBR modulated the expression of these proteins, inhibited ERS, and exerted vascular protective effects (Figure 1).

### 2.2 Inhibition of cell apoptosis

Apoptosis is a genetically controlled, programmed cell death process that is necessary for maintaining body homeostasis under both physiological and pathological conditions. The main apoptotic pathways include the intrinsic mitochondrial pathway, the extrinsic death receptor pathway, and the ERS-mediated pathway. These three pathways are interconnected through the cysteine-aspartic protease (caspase) family, with the mitochondrial pathway being the predominant route for apoptosis (Thornton et al., 2017).

In myocardial vascular endothelial cells, after receiving apoptotic signals, mitochondria alter their transmembrane structure and dissipate membrane potential, increasing the permeability of the inner membrane. At the same time, mitochondrial permeability transition pores (MPTs) form on the outer mitochondrial membrane, enabling the transport of pro-apoptotic factors such as BAX and BAK into the cytoplasm. This process facilitates the release of cytochrome C (Cyt c) and increases the release of mitochondrial small-molecule caspase activation factors (SMACs), creating conditions for apoptosis to occur (Batandier et al., 2004).

Cyt c interacts with apoptotic protease activating factor-1 (APAF-1) to form the apoptosome. The apoptosome binds with caspase-9 and activates the caspase-9 holoenzyme, which in turn cleaves caspase-3 and caspase-7, initiating the apoptotic program. SMACs bind to inhibitors of apoptosis proteins (IAPs), blocking the inhibitory effect of the X-linked inhibitor of apoptosis protein (XIAP) on caspases, thereby indirectly promoting apoptosis (Chu et al., 2021).Additionally, extrinsic apoptosis involves the activation of caspase-8. Caspase-8 mediates the cleavage of BID (a member of the BCL-2 family) into truncated BID (tBID). Truncated BID promotes the release of pro-apoptotic proteins BAX and BAK, inducing mitochondrial outer membrane permeabilization and triggering apoptosis (Chu et al., 2021).ER-mediated apoptosis is closely associated with CHOP, JNK signaling pathways, or caspase-12 (Wang Y. et al., 2020). Persistent apoptosis can lead to structural and functional damage to myocardial vascular endothelial cells, triggering endothelial dysfunction (Pang et al., 2024).

**FIGURE 2:**
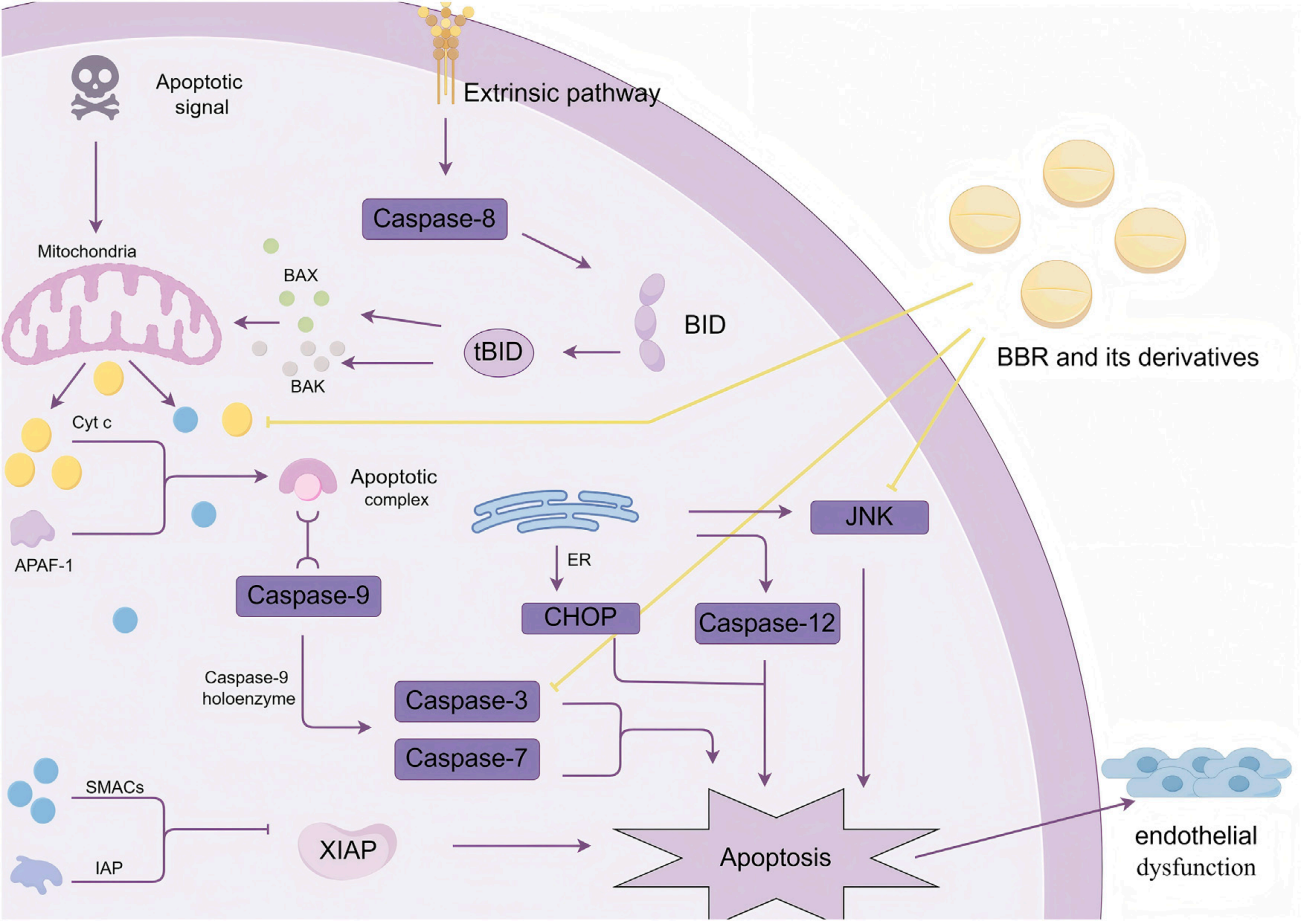
BBR and its derivatives influence the mechanism of apoptosis. They impact the regulation of apoptosis by reducing Cyt c release, inhibiting caspase-3 expression, and modulating the activation of the JNK pathway, sharp arrows (→) indicate stimulation, and blunt arrows (⊥) indicate inhibition. The figure was drawn using Figdraw.

Qin et al. (2019) showed that PA increased mitochondrial outer membrane permeability, leading to the release of Cyt c and mitochondrial dysfunction. However, pre-treatment with BBR inhibited this process. BBR reduced PA-induced caspase-3 expression and increased the expression of the anti-apoptotic protein Bcl2, thereby decreasing apoptosis. Wu L. et al. (2024) constructed a prediabetic rat model and found that BBR significantly reduced oxidative stress and apoptosis in the hippocampal tissue of rats, improving cognitive dysfunction in prediabetic rats. Xu et al. (2018) demonstrated that BBR inhibited hepatocyte apoptosis both *in vitro* and *in vivo* by reducing the release of Cyt c, Bax/Bcl-2 ratio, and caspase-3/-9 cleavage, effectively alleviating D-galactosamine (D-GalN)/LPS-induced acute liver failure. Other studies have shown that THBER and EPI N-oxide also exhibit significant anti-apoptotic effects (Wu H. et al., 2024; Nigdelioglu Dolanbay et al., 2021). Guo et al. (2021) found that human umbilical vein endothelial cells (HUVECs) and human pulmonary microvascular endothelial cells (HPMECs) treated with LPS showed increased apoptosis. However, BBR inhibited the JNK signaling pathway, promoting the survival and proliferation of endothelial cells, decreasing apoptosis rates, and improving cell survival, thus protecting vascular endothelial cells (Figure 2).

### 2.3 Reducing inflammatory response

Nuclear factor kappa-B (NF-κB) is an important nuclear transcription factor that plays a central role in the inflammatory response. The signal transduction pathway of NF-κB consists of five subunits: RelA (p65), RelB, cRel, NF-κB1 (p50), and NF-κB2 (p52). These subunits dimerize to form unique NF-κB dimers. In its resting state, the NF-κB dimers are bound to inhibitory proteins (IκB), forming an inactive heterotrimer complex that is stored in the cell. Upon stimulation by external inflammatory signals, the IκB kinase (IKK complex) induces the phosphorylation and degradation of IκB proteins, releasing the NF-κB dimers. The NF-κB dimers then translocate to the nucleus, where they bind to DNA sequences and initiate the transcription of target genes (Dorrington and Fraser, 2019).

NF-κB signaling can be activated through both classical and non-classical pathways. The classical NF-κB signaling pathway is activated by pro-inflammatory cytokines such as tumor necrosis factor (TNF-α) and interleukin-1 (IL-1), which promote the phosphorylation and degradation of IκB (especially IκBα), separating it from the p50, p65, or cRel dimers, and inducing gene transcription. The non-classical NF-κB pathway is activated by cytokines such as CD40, B cell-activating factor (BAFF), and lymphotoxin β (LT-β). Upon stimulation, the p100 protein is cleaved to form the active p52 protein, which promotes the transcriptional activation of the p52-RelB dimer (Wang et al., 2023). The classical NF-κB pathway plays an important role in regulating the levels of inflammatory factors and apoptosis-related factors, especially in vascular calcification and injury processes (Miyazaki-Anzai et al., 2024).

**FIGURE 3:**
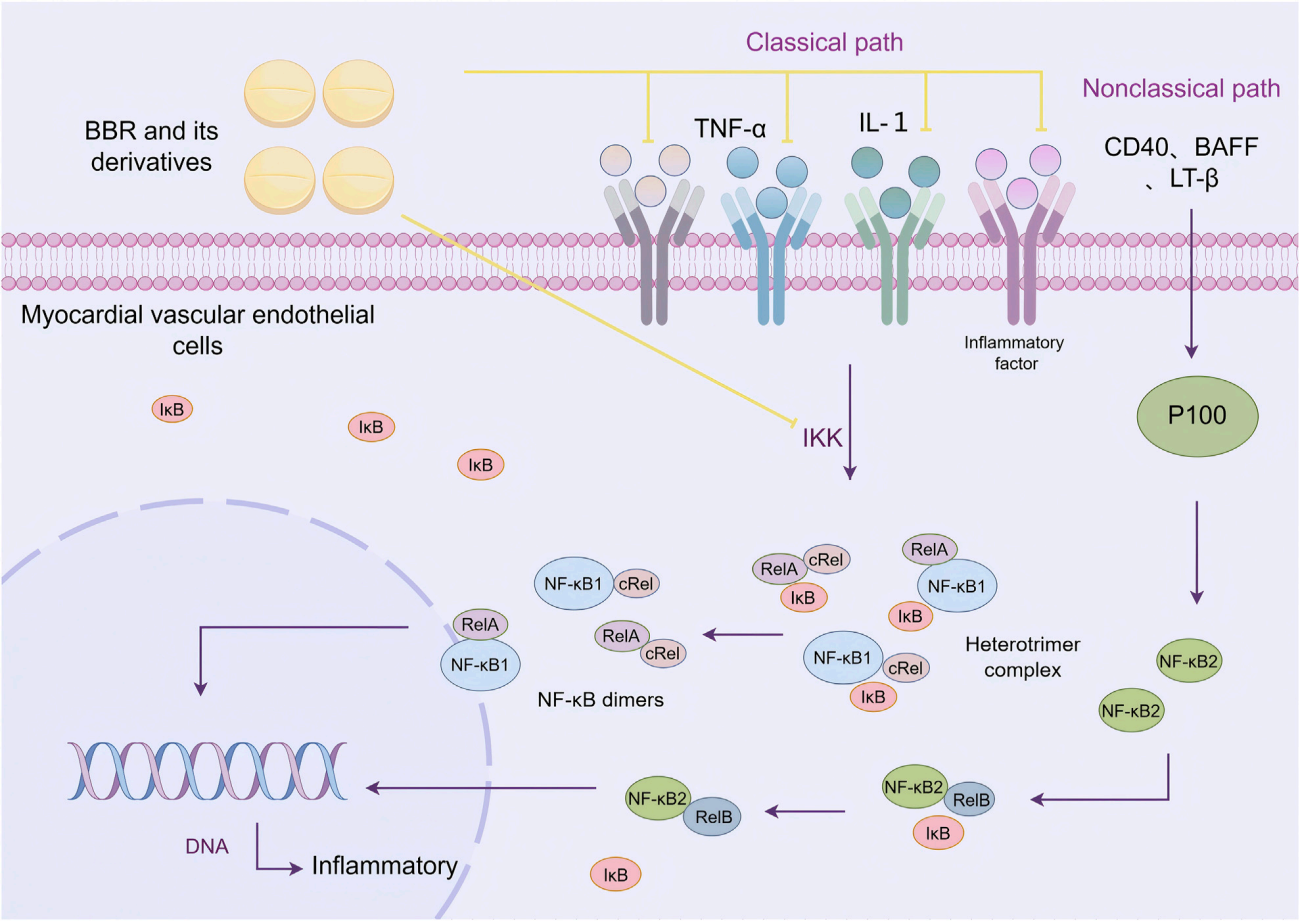
BBR and its derivatives are involved in cellular inflammatory mechanisms. They regulate cellular inflammation by inhibiting pro-inflammatory factors such as TNF-α and IL-1, and by suppressing the activation of the IKK complex, sharp arrows (→) indicate stimulation, and blunt arrows (⊥) indicate inhibition. The figure was drawn using Figdraw.

Wei et al. (2023) found that BBR can reduce the secretion of inflammatory cytokines such as IL-1β, IL-6, IL-18, and TNF-α. In EIF2AK2 gene knockout mice, the inhibitory effect of BBR was reduced, indicating that EIF2AK2 is a key target for BBR in treating inflammation. Reddi et al. (2021) constructed a LPS-induced human monocytic (THP-1) inflammatory model and found that BBR could inhibit the activation of IKKα, affect the nuclear translocation of NF-κB p65, and alleviate LPS-induced endothelial inflammation. Similarly, De et al. (2021) demonstrated that a traditional Chinese medicine formula containing BBR and EPI could block NF-κB, p38-MAPK, and ERK1/2 signaling pathways, alleviating LPS-induced inflammation. Chen L. et al. (2021) showed that BBR pretreatment reduced the expression of NF-κB and Yin Yang 1 (YY1) signaling pathways in HUVECs induced by TNF-α, decreasing the levels of cytokines and the mRNA expression of NF-κB p65 and YY1. This reduced the TNF-α-induced increase in endothelial permeability and alleviated endothelial cell damage. Other studies also suggested that BBR could inhibit the NF-κB signaling pathway, suppressing fetal bovine serum (FBS)-induced migration of human aortic smooth muscle cells (HASMC) and reducing endothelial damage (Liu et al., 2014) (Figure 3).

### 2.4 Reducing oxidative stress

Oxidative stress refers to a state of imbalance between oxidation and antioxidation within the body. This state leads to interactions between free radicals and biomolecules, increasing the expression of neutrophils and macrophages. It also causes excessive secretion of the phagocyte oxidase (Phox) complex, catalyzing the production of large amounts of oxidative intermediates, which in turn affect mitochondrial function, impair DNA repair, and damage cells.In myocardial vascular endothelial cells, oxidative stress responses are primarily mediated by reactive oxygen species (ROS). The generation of ROS is regulated by the activity of specific antioxidant enzymes such as superoxide dismutase (SOD) and catalase (CAT) (Di Meo and Venditti, 2020; Zheng et al., 2022).

Nitric oxide (NO) participates in the physiological processes of vascular dilation and constriction. ROS reacts with NO and superoxide anions (O^2−^) to form peroxynitrite (ONOO−), which reduces the bioavailability of NO and weakens vasodilation. ONOO− further affects vascular endothelial cell activity in the myocardium by participating in protein nitration reactions and mitochondrial function, exacerbating vasoconstriction.ROS can also reduce NO levels by inducing “eNOS uncoupling,” leading to long-term impairment of endothelial relaxation function and damage to myocardial vascular endothelial cell function. Moreover, ROS influence the expression of pro-inflammatory cytokines and cellular adhesion molecules such as vascular cell adhesion molecule-1 (VCAM-1) and intercellular adhesion molecule-1 (ICAM-1). Pro-inflammatory cytokines promote insulin resistance and monocyte infiltration in myocardial vascular endothelial cells, triggering chronic inflammation and endothelial damage. Increased expression of adhesion molecules induces leukocyte adhesion and enhances leukocyte extravasation into the vascular wall, impairing vascular tone and permeability (Shaito et al., 2022; Santoro et al., 2023).

**FIGURE 4:**
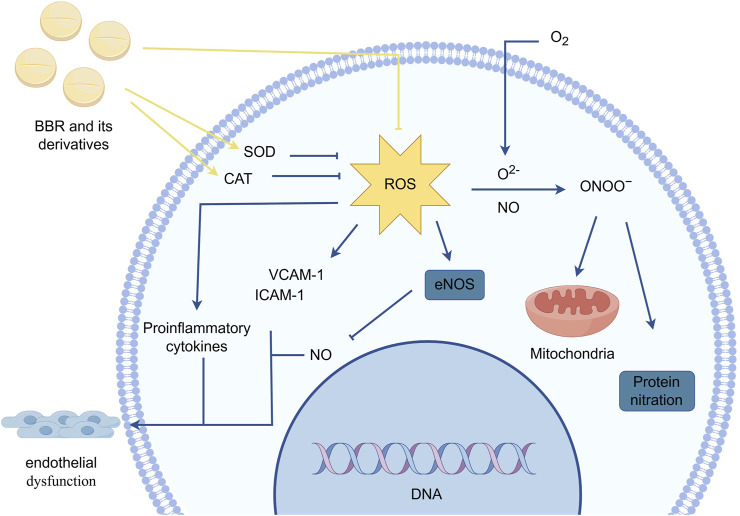
BBR and its derivatives are involved in oxidative stress mechanisms. They influence the oxidative stress response by increasing the activity of specific antioxidant enzymes such as SOD and CAT and reducing ROS production, sharp arrows (→) indicate stimulation, and blunt arrows (⊥) indicate inhibition. The figure was drawn using Figdraw.

Tian et al. (2023) found that BBR can scavenge ONOO-, NO, hydroxyl radicals (OH⋅), and Fe^2^⁺-induced oxidative stress. The half-maximal inhibitory concentrations (IC50s) of BBR for scavenging NO radicals, chelating Fe^2^⁺, and scavenging OH⋅ were 0.17 mg/mL, 0.12 mg/mL, and 0.11 mg/mL, respectively. Zhang et al. showed that BBR selectively inhibits the expression of the gp91 subunit of Phox, enhances the activity of SOD and CAT, and effectively suppresses ROS generation in cells, reducing oxidative stress-induced cytotoxicity and cellular damage (Zhang et al., 2021; Li et al., 2017a).Furthermore, Cheng et al. (Cheng et al., 2013) demonstrated that BBR could reverse excessive ROS generation induced by circulating endothelial microparticles (EMP) in HUVECs. Other studies in rat myocardial hypoxia injury models showed that H2O2 promotes ROS generation, while BBR derivatives such as raisanberine (RS) can reduce ROS production by inhibiting NADPH oxidase (NOX) activity in myocardial cells (Gao et al., 2012) (Figure 4).

### 2.5 Autophagy-related effects

Autophagy is a lysosome-dependent regulatory mechanism that removes misfolded or aggregated proteins, damaged organelles, and pathogens to maintain cellular homeostasis. It can be categorized into three forms: macroautophagy, microautophagy, and chaperone-mediated autophagy (CMA). Among these, macroautophagy is the predominant form involved in cardiovascular endothelial damage-related autophagy (Yamamoto and Matsui, 2024). Impaired autophagy in myocardial vascular endothelial cells is closely associated with various vascular injuries, including atherosclerosis. When myocardial vascular endothelial cells are damaged by factors such as ischemia-reperfusion, oxidative stress, or inflammatory infections, macroautophagy is triggered. Under normal conditions, autophagy begins with the activation of autophagy-inducing factors (e.g., TNF-α, ROS, IL), which activate the ULK1 complex and various ATG proteins. These proteins localize to the pre-autophagosomal site, where a phagophore (isolation membrane) forms in the cytoplasm. This membrane structure progressively expands and extends, engulfing cytoplasmic components to form an autophagosome. The autophagosome then fuses with the lysosome, where lysosomal hydrolases degrade its contents, releasing nutrients and metabolic byproducts for cellular reuse (Glick et al., 2010).Under basal conditions, autophagy is expressed at low levels in vascular endothelial cells, which helps reduce the release of pro-apoptotic factors, eliminate abnormal cells, enhance cellular energy levels, and maintain normal cardiovascular function (Liu et al., 2023). Based on this, Lin et al. (Lin et al., 2022) concluded that increasing basal autophagy and preventing age-dependent declines in autophagy levels are potential targets for mitigating vascular endothelial inflammation, combating atherosclerosis, and maintaining endothelial function.

Dysregulated autophagy is a critical factor in myocardial vascular endothelial cell damage, including both autophagy suppression and excessive autophagy. The traditional autophagy pathway is the AMPK pathway (Li and Chen, 2019).As a key upstream regulator of autophagy, AMPK promotes autophagy by directly phosphorylating autophagy-related proteins or inhibiting negative regulators of autophagy (Sachdev and Lotze, 2017). Specifically, AMPK activates ULK1 by phosphorylating its Ser317 and Ser777 sites and facilitates autophagosome formation by phosphorylating ATG14 at the Ser29 site (Lu et al., 2023). It can also directly phosphorylate Beclin1 and Vps34 to activate autophagy (Wang et al., 2019). AMPK additionally inhibits mTOR activation or rapamycin-induced mTORC1 phosphorylation, thereby inhibiting autophagy activation (Chun and Kim, 2021). ULK1 is a key kinase in the initiation stage of autophagy, and its phosphorylation can block the physiological transmission of autophagy signaling pathways. The most fundamental mechanism by which AMPK regulates cell growth is through the inhibition of the mTORC1 pathway (Mihaylova and Shaw, 2011). When nutrients are abundant, mTORC1 inhibits autophagy by phosphorylating substrates such as ULK1, including phosphorylation at sites like Ser555 and Ser757 on ULK1, thereby preventing AMPK from activating ULK1 (Ran et al., 2020). mTORC1 also indirectly inhibits autophagy by regulating other autophagy-related proteins and complexes, such as the PI3KC3 complex I (involved in the formation of autophagosomes) and TFEB (a major transcriptional regulator of lysosomal biogenesis and autophagy-related genes) (Wirth et al., 2013; Nnah et al., 2019). While, when nutrients are insufficient or lack of stress, the activity of mTORC1 decreases and the autophagy process is activated to clean up intracellular wastes and damaged organelles.JNK autophagy pathway is one of the other important mechanisms in the regulation of cellular autophagy.Under stress, JNK activates and phosphorylates Bcl-2/Bcl-xL, which destroys the complex of Bcl-2/Bcl-xL and Beclin1 and makes the Beclin1 is released, and the free Beclin1 binds to hVps34 and PI3K, forming Beclin1-hVps34-PI3K complex, which activates PI3KIII and induces autophagy (Zhou et al., 2015).

In addition to the traditional pathways mentioned above, an increasing number of “atypical autophagy pathways” in myocardial vascular endothelial cells have been identified in recent years, such as the mitochondrial PINK1/Parkin-mediated mitophagy pathway (Zhao et al., 2024). Although the mitochondrial content in vascular endothelial cells (ECs) is significantly lower than in cardiomyocytes, under the influence of reactive oxygen species (ROS), dysfunctional mitochondria fail to be effectively cleared, ultimately resulting in endothelial cell damage. Experiments by Zhang et al. (2023) demonstrated that knockdown of CD44 can suppress the senescent phenotype of vascular endothelial cells, while overexpression of CD44 directly induces premature senescence in young vascular endothelial cells. CD44 primarily regulates the autophagy process through its intracellular domain (CD44ICD), and overexpression of CD44ICD inhibits autophagic activity in mouse vascular endothelium, thereby accelerating vascular aging. Ma et al. (2023) demonstrated that the Wnt/β-catenin pathway compensates for molecular damage caused by the imbalance between autophagy and apoptosis. Similarly, Wan et al. (2020) revealed that the cGAS-STING pathway can induce autophagy. When cGAS recognizes and binds cytoplasmic DNA, it generates the secondary messenger cGAMP, which activates STING, a critical pathway in antiviral responses of myocardial endothelial cells.Moreover, the role of TFEB, a central regulator of autophagy and lysosomal gene expression, has gained increasing attention. TFEB promotes the expression of autophagy-related genes, facilitates autophagosome formation, and regulates lysosomal biogenesis. It interacts with mTORC1, and the inactivation of mTORC1 drives TFEB dephosphorylation and nuclear translocation, initiating the transcription of target genes to enhance autophagy (Franco-Juárez et al., 2022).The interplay between ferroptosis and autophagy in myocardial vascular endothelial cells has become a hot topic in recent years. During Erastin-induced ferroptosis, lipid peroxidation products and oxidative stress generated can influence the formation and degradation of autophagosomes, leading to autophagic defects (Chen X. et al., 2021).

**FIGURE 5:**
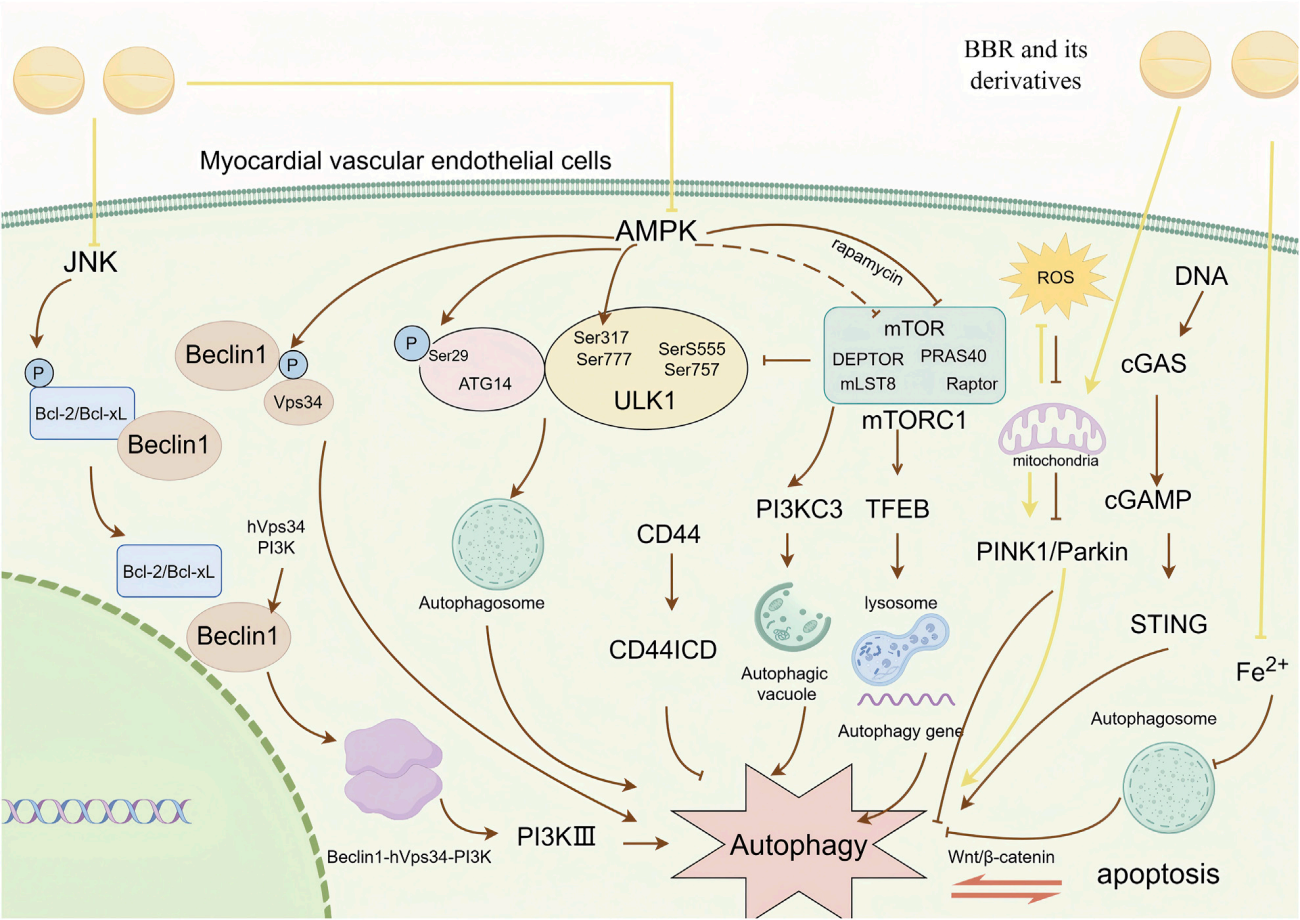
BBR and its derivatives are involved in autophagy mechanisms. They influence the autophagy by inhibiting the JNK and AMPK signaling pathways, enhancing the mitochondrial PINK1/Parkin signaling pathway, and influencing ferroptosis regulatory processes, sharp arrows (→) indicate stimulation, and blunt arrows (⊥) indicate inhibition. The figure was drawn using Figdraw.

Based on the overview of pathways involved in autophagic impairment, we summarise that BBR’s therapeutic approach should focus on targeting these pathways. For instance, HU’s research demonstrates that BBR inhibits excessive autophagy through AMPK, maintains mitochondrial function, improves cellular energy supply and physiological redox state, and reduces myocardial vascular endothelial cell damage caused by MI/RI (Hu et al., 2024). GUO’s study (Guo et al., 2021) confirms that BBR blocks the JNK pathway and inhibits LPS-induced autophagy in vascular endothelial cells. Specifically, it reduces Beclin-1 levels, downregulates the LC3-II/LC3-I ratio, and upregulates p62 protein expression, thereby mitigating LPS-induced cellular autophagy. Kou et al. (2017) discovered that berberine (BBR) reduces the phosphorylation levels of Akt and mTOR in the PI3K/Akt/mTOR signaling pathway. Akt and mTOR, as key molecules responsible for inducing autophagy, exhibit phosphorylation that signifies abnormal autophagy. Furthermore, Kou’s research showed that BBR promotes autophagy in vascular macrophages, inhibits it in foam cells, accelerates cholesterol efflux, and thereby exerts positive effects in combating atherosclerosis.ZHOU’s experiments (Zhou et al., 2024) demonstrated that BBR activates the SIRT6-AMPK-FOXO3a signaling pathway, enhancing mitochondrial biogenesis and PINK1/Parkin-mediated mitophagy. This activation reduces oxidative stress-induced damage and preserves mitochondrial function. These findings provide significant evidence that BBR has a definitive protective effect on the mitochondria of myocardial vascular endothelial cells.YANG’s experiments in H9c2 cardiomyocytes revealed that BBR mitigates cell viability loss induced by erastin and RSL3, two ferroptosis activators. Additionally, BBR reduces the accumulation of ROS and lipid peroxidation caused by ferroptosis, thereby maintaining endothelial cell activity (Yang et al., 2022). CHENG’s experiments (Cheng et al., 2023) demonstrated that BBR reverses mTORC1-regulated macrophage polarization by activating AMPK to reprogram glycolysis.This function is indirectly achieved through the phosphorylation of raptor, a key component of mTORC1, and TSC2, a tumor suppressor protein that inhibits mTORC1 activity. Liu et al. (2020) experiments also demonstrated that BBR induces autophagic cell death in a dose-dependent manner by inactivating the AKT/mTORC1 signaling pathway. The commonality between these two findings confirms that mTORC1 is a potential target through which BBR regulates autophagy in ECs. Wang et al. (2016) experiment also confirmed that BBR, by interfering with the AMPK/mTOR/ULK1 pathway, altered the cellular metabolic state, resulting in impaired high autophagic flux and glycolytic capacity in cells (Figure 5).

### 2.6 Inhibition of platelet adhesion and aggregation

Platelets, being one of the key cells implicated in endothelial damage, play a crucial role in maintaining the integrity of blood vessels and the repair of vascular injuries. Myocardial vascular endothelial cells induce vasoconstriction, reducing the vessel diameter and causing turbulence at the vessel wall, thereby providing the initial conditions for platelet adhesion. Damaged myocardial blood vessel walls release tissue factors, which directly activate the blood coagulation system. Under the influence of von Willebrand factor (VWF), platelets adhere to the extracellular matrix of the myocardial vascular endothelium, forming the first thin layer over the myocardial endothelial defect. The binding of glycoprotein VI (GPVI) to collagen activates the platelet collagen receptor α2β1 (GPIa/IIa), further enhancing platelet adhesion. The increased platelet adhesion is a prerequisite for platelet activation. Moreover, agonists such as ADP released from damaged red blood cells and thrombin generated during coagulation can trigger platelet activation via their G protein-coupled receptors (GPCRs), leading to an increase in intracellular Ca^2^⁺ concentrations, membrane phospholipid release, and activation of specific signaling pathways involved in thrombus formation. Platelet activation causes a conformational change in the fibrinogen receptor integrin αIIbβ3 (GPIIb/IIIa) on the platelet surface, facilitating platelet aggregation. Thrombus formation then activates the coagulation cascade, leading to the conversion of plasma protein fibrinogen into fibrin, which polymerizes into a fibrin network and forms a blood clot with aggregated platelets to complete hemostasis (Neubauer and Zieger, 2022). However, sustained and abnormal platelet hyperactivity and a procoagulant state result in enhanced platelet adhesion, activation, and aggregation, triggering pro-inflammatory responses, inducing thrombosis, causing myocardial endothelial vascular cell damage, and contributing to cardiovascular diseases (Higashikuni et al., 2021).

**FIGURE 6:**
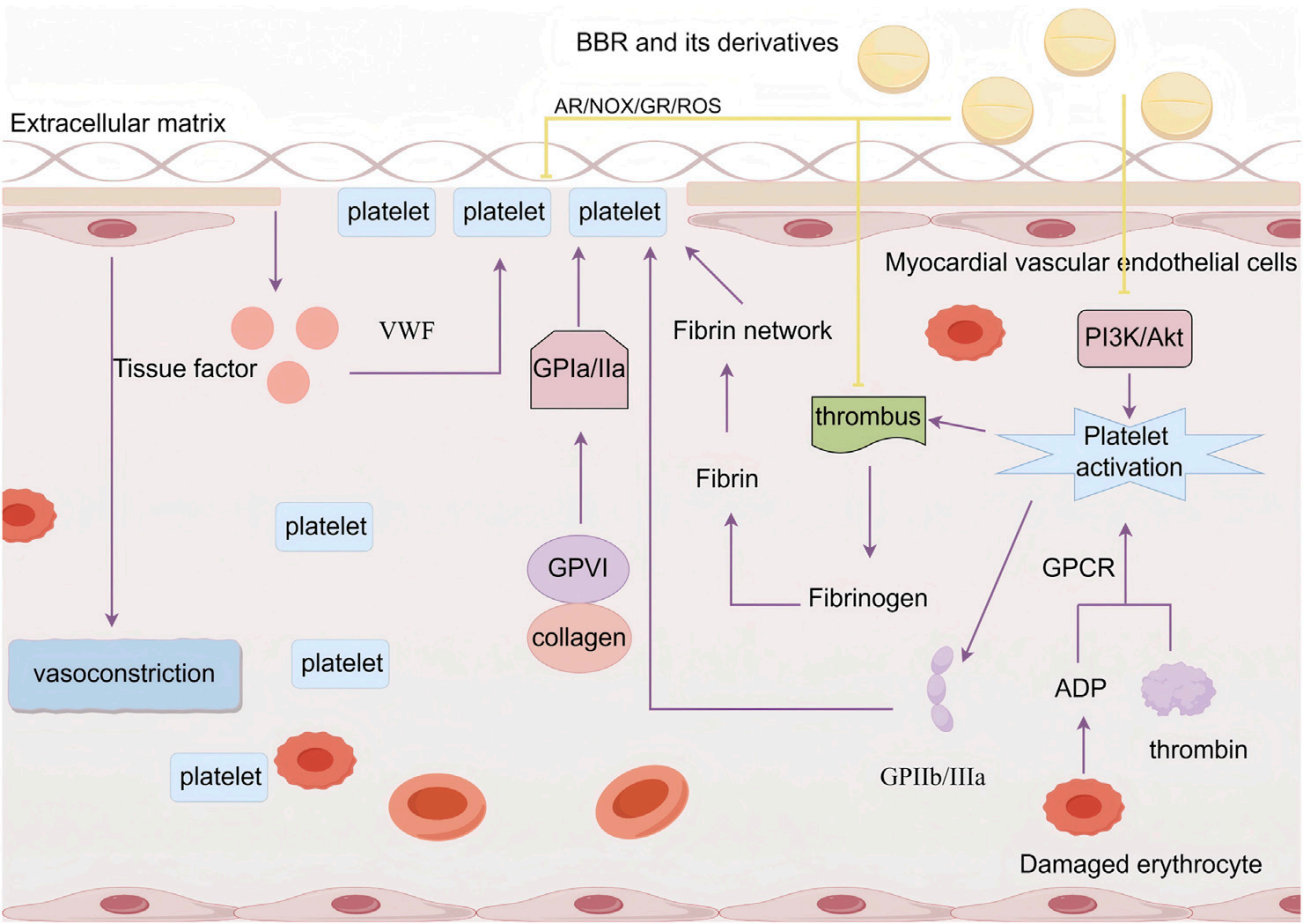
BBR and its derivatives are involved in platelet adhesion and aggregation mechanisms. They influence the platelet adhesion and aggregation by inhibiting the PI3K/Akt pathway to suppress platelet activation, regulating AR/NOX/GR activity, reducing ROS levels, and affecting thrombus formation, sharp arrows (→) indicate stimulation, and blunt arrows (⊥) indicate inhibition. The figure was drawn using Figdraw.

Wei et al. (2022) used a carrageenan-induced mouse thrombus model and found that compared to the control group, the tail vessels at 2 cm and 4 cm positions in carrageenan-treated mice were almost completely occluded by thrombi, while the distal 6 cm vessels showed about 60% narrowing. When treated with 227.5 mg/kg, 445 mg/kg, and 910 mg/kg doses of a traditional Chinese medicine compound, Gegenqinlian tablets (containing BBR), thrombus formation at multiple sites was significantly inhibited. This also alleviated the degree of vessel narrowing, increased blood flow, and reduced plasma levels of TNF-α. Wei et al. further established an *in vitro* thrombus model using LPS-induced HUVECs and found that BBR and its derivatives could reduce platelet adhesion to LPS-stimulated HUVECs, lower the expression of TNF-α, NF-κB, and NLR family pyrin domain containing 3 (NLRP3), thereby inhibiting thrombus formation and reducing MVEI. Wang C. et al. (2021), using a mouse thrombus model, found that BBR and its derivative “Berberrubine” suppressed platelet activation and aggregation by inhibiting the PI3K/Akt pathway. Paul et al. (2019) showed that BBR could inhibit platelet aggregation in a high glucose (HG)-treated human platelet model by modulating the activities of aldose reductase (AR), NOX, and glutathione reductase (GR), as well as reducing the production of ROS and superoxides. Wang X. et al. (2017), through structural, functional, and binding studies on BBR, discovered that BBR is a direct thrombin inhibitor and can inhibit thrombin-induced platelet aggregation, providing direct evidence for the anticoagulant mechanism of BBR (Figure 6).

### 2.7 Regulation of metabolic products of gut microbiota

The gut microbiota is a crucial component of the human body and plays a vital role in the development and progression of cardiovascular diseases. The gut microbiota exerts its effects on MVEI and repair primarily through the release of metabolic products such as trimethylamine-N-oxide (TMAO), short-chain fatty acids (SCFAs), bile acids (BA), phenylethylamine glutamine (PAGln), and endotoxins. This section focuses on the impact of these metabolic products on myocardial vascular endothelial cells and heart function.

TMAO, one of the metabolic products produced by gut microbiota, is widely distributed in vascular-rich organs such as the heart and kidneys. Previous studies (Messenger et al., 2013) have shown that TMAO participates in various physiological processes, such as activating platelet aggregation, inducing inflammatory responses, increasing foam cell generation, affecting cholesterol transport, and promoting vascular calcification and vascular damage. The levels of TMAO are positively correlated with the degree of atherosclerosis. Zhang X. et al. (2020) demonstrated using calcium content assays that TMAO promotes calcium/phosphate-induced vascular smooth muscle cell (VSMC) calcification in both rats and humans, exhibiting a dose-dependent effect. TMAO promotes vascular calcification through activation of the NLRP3 inflammasome and NF-κB signaling. In an animal study (Zhang et al., 2024), TMAO suppressed Ca^2^⁺influx in mouse vascular endothelial cells through the TRPV4-NO pathway, indicating that TMAO affects endothelial ion exchange and impairs vascular dilation function. A study (Seldin et al., 2016) found that TMAO plays a role in vascular endothelial leukocyte recruitment, which induces inflammation and counteracts the permeability of vascular endothelial cell membranes. Ke et al. (2018) observed that TMAO levels increase with age by analyzing its content in individuals from different age groups and mice of varying ages. The study further revealed that TMAO contributes to vascular endothelial cell senescence and death, as evidenced by reduced cell proliferation, G0/G1 phase arrest in the cell cycle, and impaired cell migration. These effects are likely mediated through the inhibition of SIRT1 expression and the activation of the p53/P21/Rb signaling pathway.

Certain subtypes of SCFAs not only participate in the intestinal barrier function but also play a role in repairing MVEI. SCFAs are ligands for HDAC inhibitors, stimulating monocytes and neutrophils through the HDAC pathway, leading to decreased expression of NF-κB, interleukins, and TNF-α. They may also promote the activation of inflammasomes in intestinal epithelial cells (IECs) to produce anti-inflammatory factors. For instance, in an animal experiment by Yan et al. (2022), increasing SCFAs (especially propionate) significantly improved gut barrier function and suppressed the release of inflammatory factors. This effect may protect cardiovascular endothelial cells and inhibit vascular calcification through the “intestinal tract-vessel axis.” Hong et al. (2024) found that propionate counteracted the adverse effects of ox-LDL on vascular endothelial cells and also suppressed endoplasmic reticulum stress, thereby providing protection for cardiovascular endothelial cells. In contrast, acetate, when bound to coenzyme A, becomes a central molecule in carbohydrate and fat metabolism. Abnormal acetate levels often lead to lipid metabolism disorders, with excessive lipids adhering to myocardial vascular endothelium, contributing to atherosclerotic plaque formation. It is generally believed that butyrate inhibits the expression of inflammatory factors, reducing the inflammatory response and vascular calcification in endothelial cells. However, contradictory findings from Zhong’s study (Zhong et al., 2022) revealed that butyrate induced rapid activation of NF-κB, Wnt, and Akt signaling in VSMCs. This dual effect—HDAC inhibition and NF-κB activation—accelerated vascular calcification, which appears to conflict with existing conclusions. Another animal experiment (Brandsma et al., 2019) introduced pro-inflammatory Casp1−/− microbiota into Ldlr−/− mice and found that this microbiota increased systemic inflammation, reduced SCFA levels in feces, and accelerated MVEI and atherosclerotic plaque progression.

The most important role of BA is their effect on lipids, particularly in regulating cholesterol uptake and maintaining lipid metabolic homeostasis. This regulation is primarily mediated by the activation of Farnesoid X receptor (FXR) and G protein-coupled receptor 5 (TGR5) receptors by bile acids (Chiang and Ferrell, 2020). When BA secretion is dysregulated, excess lipid particles can enter the bloodstream and adhere to the vascular endothelium, leading to MVEI.An animal study (Meissner et al., 2013) demonstrated that bile acid sequestrants (BAS) effectively reduced bile cholesterol secretion and plasma cholesterol levels in bile, while increasing fecal neutral cholesterol levels in male Ldlr-deficient mice. This study further showed that BAS mitigated MVEI and reduced the extent of atherosclerosis in these mice.

The gut microbiota-derived metabolic product PAGln promotes myocardial vascular endothelial damage by activating platelets, triggering inflammatory responses, and other mechanisms. Specifically, PAGln mediates cell events through G protein-coupled receptors, such as α2A, α2B, and β2-adrenergic receptors. Similar to catecholamines, PAGln exerts its effects through adrenergic receptors and GCPR-mediated cellular responses, enhancing platelet activation and thrombosis risk in whole blood, isolated platelets, and arterial injury animal models (Nemet et al., 2020). In an *in vitro* study of PAGln on HL-1 cells (Fang et al., 2022), PAGln was found to increase ROS production, enhance the activation of CaMKII and RyR2, promote apoptosis, and reduce cell viability. These findings suggest that PAGln may play a role in the processes ofoxidative stress-induced and apoptosis damage.

Endotoxins, found on the cell membranes of Gram-negative bacteria, directly damage vascular endothelial cells, leading to endothelial degeneration, necrosis, and detachment, thereby compromising the integrity of the vascular endothelium. Laboratory studies (Stoll et al., 2004) have confirmed that even very low levels of endotoxin (0.1 ng/mL) significantly damage human coronary smooth muscle cells, releasing more pro-inflammatory cytokines and exacerbating the inflammatory response in the vascular endothelium. Higher levels of endotoxin lead to more severe damage to vascular endothelial cells, including increased production of cytokines, reactive oxygen species (ROS), reactive nitrogen species, prostaglandins, and tissue factor. These changes contribute to the upregulation of adhesion molecule expression and the loss of vascular endothelial cell monolayer integrity and barrier function (Bannerman and Goldblum, 1999).The Toll-like receptor (TLR) family, particularly TLR4, plays a key role in endotoxin-induced vascular endothelial damage. This pathway has been widely recognized for its role in vascular injury. A review published in 2006 (Stoll et al., 2006) summarized direct evidence of TLR4 involvement in vascular injury, showing that TLR4-deficient mice exhibit larger myocardial infarction areas after coronary artery ligation and reperfusion, and TLR4 participates in lipid metabolism and inflammation. In Stoll LL’s study (Hong et al., 2024), it was further verified that serum lipoproteins, especially HDL, modulate the inflammatory response induced by endotoxins, providing a potential target for treatment.

The effective intervention of BBR and its derivatives helps restore the balance of intestinal microbiota, enhance the protective barrier function, inhibit excessive immune responses and inflammation, and regulate the release of metabolites, thereby exerting vasoprotective effects. Xie et al. (2021) found that BBR significantly reduced levels of choline trimethylamine cleavage enzyme (CutC) in the cecal contents by promoting the growth of *Lactobacillus* spp., which reshaped the intestinal microbiota structure in rats. This reduction in CutC activity led to decreased production of TMAO and TMA induced by a choline-rich diet, thereby lowering the potential risk of arterial thrombosis. Similarly, Qu et al. (2024) demonstrated that BBR exerted its protective effects on vascular endothelial cells and thrombosis by inhibiting CutC enzyme activity and reducing TMAO production. Several studies (Wang et al., 2024a; Li et al., 2021; Wu et al., 2020) have shown that different concentrations of BBR can alter the intestinal microbiota composition in humans and mice, particularly by increasing the abundance of Blautia, Roseburia, Turicibacter, Allobaculum, and Alistipes, which are associated with favorable anti-inflammatory effects through SCFA production and lipid metabolism. These changes also affected the abundance of CutC/D enzymes or cutC/cntA genes, inhibiting the synthesis of TMAO precursors, lipid metabolism, and vascular inflammatory responses. Wang’s study (Wang et al., 2024b) further demonstrated that BBR ameliorated TMAO-induced ERS activation and vascular dysfunction, providing a protective effect on vascular endothelial cells.

BBR’s effects on SCFAs are also noteworthy. In a study using direct-injection GC detection and real-time polymerase chain reaction (RT-PCR) (Wang LL. et al., 2017), the researchers demonstrated that after culturing intestinal bacteria with different concentrations of BBR for 24 h, SCFA concentrations (mainly acetate, propionate, and butyrate) were significantly higher compared to bacteria cultured in saline. Moreover, the key enzymes involved in SCFA synthesis (ACK, MMD, and BUT) showed increased gene expression, with a positive correlation to BBR concentration.

Another study (Chen et al., 2020) demonstrated that BBR effectively increased the abundance of SCFA-producing bacteria, such as Alloprevotella, Flavonifractor, and Oscillibacter. Additionally, BBR was found to regulate glucose metabolism, SCFA metabolism, and amino acid metabolism, as indicated by metabolic profiling data. In a study examining colonic mitochondrial function in diet-induced obese (DIO) mice (Sun et al., 2018), it was observed that the colonic microenvironment in these mice was dysregulated, characterized by malformed, ruptured, necrotic, and dysfunctional mitochondria, along with increased apoptosis and a decrease in SCFAs. However, BBR intervention, either directly or indirectly, reversed these adverse effects, restoring mitochondrial function and increasing SCFA production.

Cholesterol utilization is closely linked to BA content, and BA secretion is regulated by BBR. Several studies (Sun et al., 2023; Tian et al., 2019) have demonstrated that BBR ameliorates DSS-induced BA imbalance in the intestine and liver, enhances the levels of conjugated and secondary BA in the gastrointestinal tract, and repairs the intestinal barrier via the FXR and TGR5 pathways. Interestingly, Wang’s study (Wang Q. et al., 2024) found that BBR specifically inhibited the *Clostridium* genera, reducing the levels of secondary BA and the BA efflux index in the enterohepatic circulation, while also inhibiting cholesterol absorption. In another study (Li XY. et al., 2015), BBR promoted the excretion of cholesterol from the liver to the bile in hyperlipidemic hamsters, simultaneously lowering blood levels of TC, TG, and LDL-C, thereby reducing the lipid-induced risk to vascular endothelium.

There are relatively few studies on the inhibitory effects of BBR on endotoxin, but all of them focus on the idea that the key to endotoxin resistance is the prevention of endotoxin-induced cellular inflammation. Some studies suggest that BBR’s endotoxin-resistant function is mediated through the AMPK and Nrf2 pathways (Mo et al., 2014), while others propose that BBR administration significantly reduces high-fat diet (HFD)-induced endotoxin levels, intestinal fatty acid binding protein, and tumor necrosis factor-α in rats (Li et al., 2017b).

However, we have not found direct or indirect evidence that BBR acts on PAGln or its precursors and transformants to exert endothelial protective effects on the myocardial vasculature. This mechanism warrants further investigation.

**FIGURE 7:**
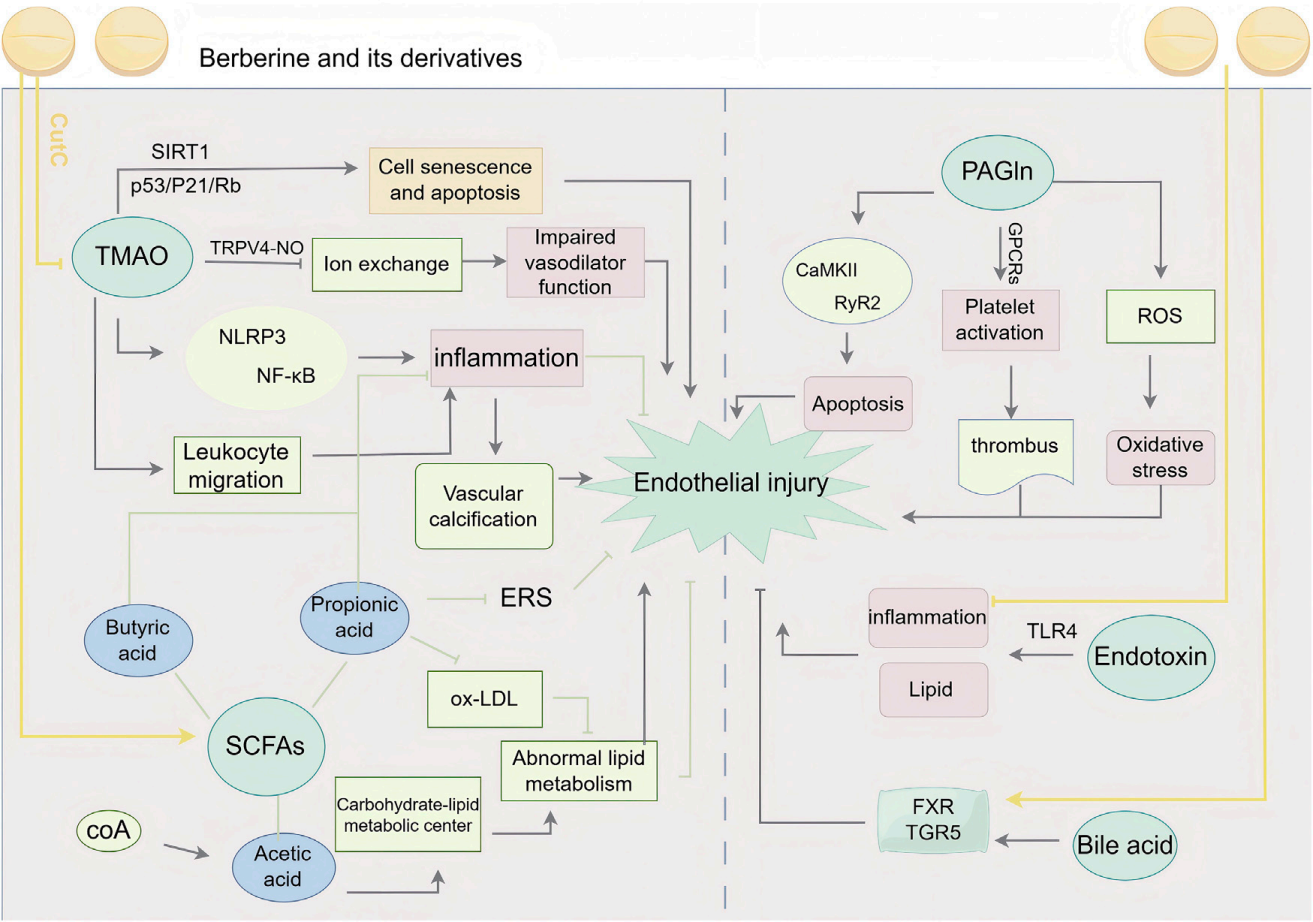
BBR and its derivatives are involved in gut microbiota mechanisms. They influence the gut microbiota by inhibiting CutC to reduce TMAO production, promoting SCFAs generation; enhancing FXR and TGR5 activity; mitigating endotoxin-induced inflammation, sharp arrows (→) indicate stimulation, and blunt arrows (⊥) indicate inhibition. The figure was drawn using Figdraw.

In conclusion, intestinal flora play a role in myocardial vascular endothelial injury, either directly or indirectly, through mechanisms such as changes in abundance, barrier damage, immune response, inflammation induction, or the release of metabolites like enteric toxins and short-chain fatty acids. BBR and their derivatives are effective interventions that can repair intestinal bacterial population disorders and protective barriers, inhibit excessive immune responses and inflammation, and control the release of these metabolites to exert vascular protection (Figure 7).

### 2.8 Schematic diagram of the mechanism of BBR in repairing myocardial vascular endothelial injury

**FIGURE 8:**
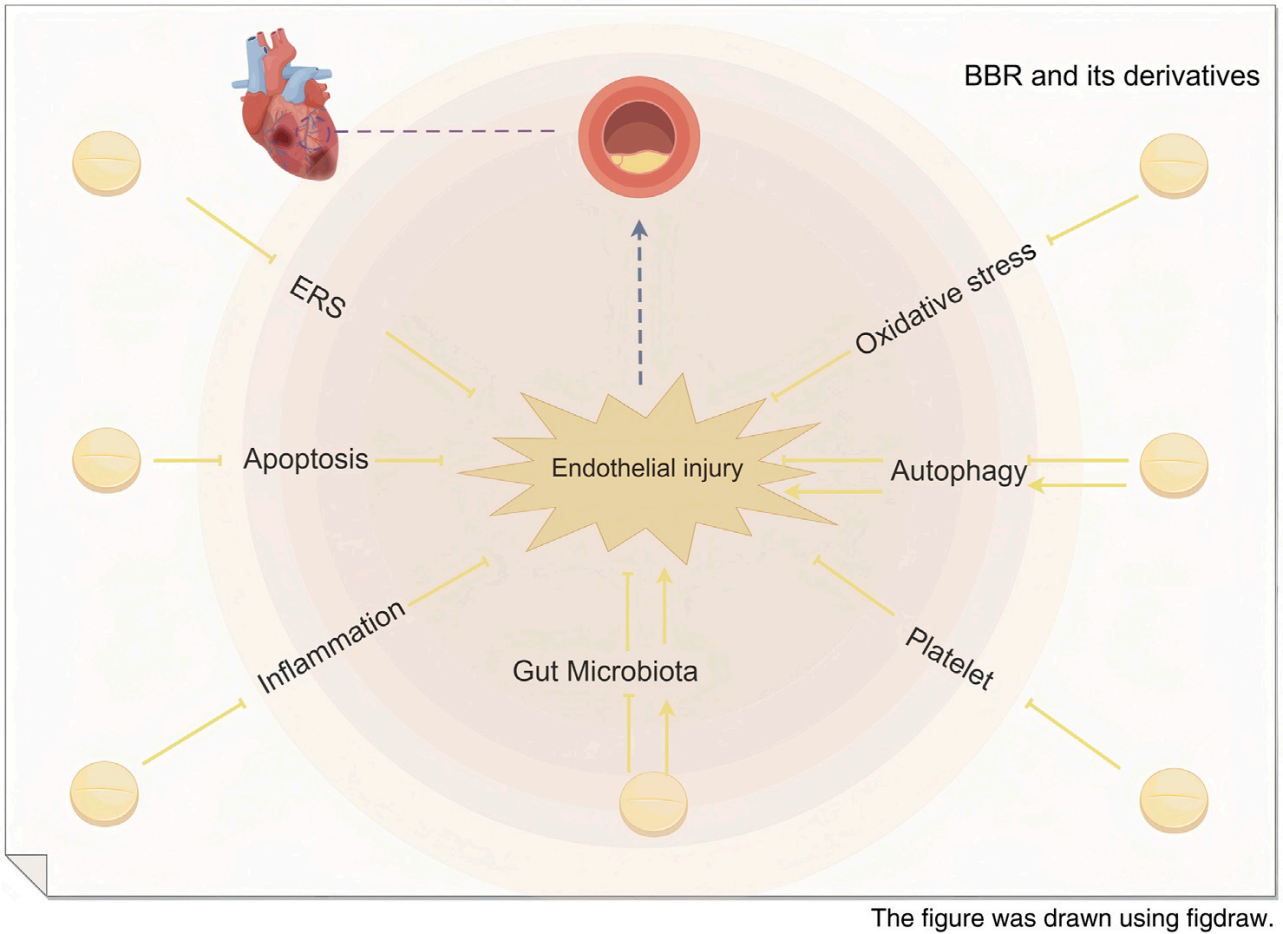
Schematic diagram of the mechanism of BBR in repairing myocardial vascular endothelial injury.

Based on the aforementioned research, the effects of BBR and its derivatives on myocardial vascular endothelial injury primarily focus on the regulation of endoplasmic reticulum stress, inhibition of cell apoptosis, reduction of inflammatory responses, alleviation of oxidative stress, modulation of autophagy-related effects, suppression of platelet adhesion and aggregation, and regulation of gut microbiota-derived metabolic products, as illustrated in Figure 8.

## 3 Major pathways and targets of BBR and its derivatives on myocardial vascular endothelial cells in the last decade

BBR and its derivatives play a significant role in repairing myocardial vascular endothelial injury. To this end, we have summarized the major pathways and targets of BBR and its derivatives on myocardial vascular endothelial cells over the past decade, as shown in Table 1.

**TABLE 1:** Major pathways and targets of BBR and its derivatives on myocardial vascular endothelial cells in the last decade

Experimental model	Medicine	A target or pathway	Effect	Ref
Apolipoprotein E-deficient mice (ApoE−/−)	8- cetylberberine (8-BBR-C16) (15 mg/kg/d)	inflammatory and oxidative markers, Especially the inhibition of the translocation of NF-κB to the nucleus	The levels of serum IL-1β, TNF-α, NF-κBp65, i-NOS, ICAM-1, IL-6 mRNA and NF-κBp65 in aorta were decreased, and the expression of i-κBα in cytoplasm was increased	Feng et al. (2017a)
Gut microbiota,HFD-fed hamsters,and patients treated with rosuvastatin plus aspirin, or clopidogrel sulfate or ticagrelor	BBR(0.5 g, bid)	Down-regulating Choline-TMA-TMAO production pathway	treat atherosclerosis	Ma et al. (2022)
Diabetic rats	BBR (50、100 and 200 mg/kg/d)	By activation of BKCa channel in VSMCs	provide a combinational therapy for controlling hyperglycemia and blood pressure	Ma et al. (2017)
Zebrafish embryos	BBR(0.01–0.75 mM)	enos/vegf signal channel	by modulating angiogenesis	Nathan et al. (2024)
spontaneously hypertensive rats	BBR(50 mg/kg.d)	endothelial microparticles (EMPs) and endothelial progenitor cells (EPCs)	BBR treatment in SHRs partly reduced the blood pressure and circulating EMPs, and augmented EPC numbers and CFUs	Zhang et al. (2020b)
cultured cells and rats	BBR(50 mg/kg.d)	Vascular smooth muscle cells (VSMCs)	berberine induces direct vasorelaxation to lower BP and reduces vascular stiffness in aged mice through suppression of TRPV4	Wang et al. (2015)
apolipoprotein E-deficient (ApoE−/−) mice	BBR(78 mg/kg.d、156 mg/kg.d	Many biological pathways, such as mitochondrial dysfunction, fatty acid β-oxidation I, FXR/RXR activation, and et al	BBR reduced serum lipid levels, antagonized hepatic lipid accumulation, improved intima-media thickening, and alleviated atherosclerotic lesions	Tan et al. (2020)
Mouse VSMCs	BBR(100 µM)	PDI-endoplasmic reticulum stress system, caspase-3 and caspase-12	berberine inhibits the PDI-endoplasmic reticulum stress system, thereby attenuating the simultaneous increase of VSMC proliferation and apoptosis in response to mechanical stretch	Wang et al. (2020b)
KD-induced human coronary artery endothelial cell (HCAECs)	BBR(20 μM)	Oxidative stress and ERs	BBR inhibited the apoptosis of HCAECs, arrested the cell cycle at G0/G1 phase and protected HCAECs from injury by inhibiting the expression of THBD, vWF and EDN1	Xu et al. (2020)
HFD-fed mice	BBR(0.5 g/L)	Modulation of gut microbiota	Anti-atherosclerotic and metabolic protective effects	Zhu et al. (2018)
LPS -induced inflammatory vascular injury in mice and mouse microvascular endothelial cells (MECs)	BBR(50, 100, or 200 mg/kg/d)	Inhibited endothelial NLRP3 inflammasome activation and Ca2+ influx	Target improved calcium signaling and endothelial NLRP3 inflammasome to reduce inflammatory damage to blood vessels	Dai et al. (2022)
Rats with metabolic syndrome	BBR(50 mg/kg/d)	p38 MAPK, ATF-2 and MMP-2	inhibit p38 MAPK activation, ATF-2 phosphorylation, and MMP-2 expression, reduce the secretion of inflammatory factors and improve vascular remodeling	Li et al. (2015b)
Vascular smooth muscle cells of thoracic aorta in T2DM rats	BBR(6.25, 12.5, 25, 50 and 100 μg/mL)	Inhibits inflammation and interferes with calcium-activated potassium (BKCa) channels	Decreased the levels of inflammatory factors such as IL-6, TNF-α, TGF-β1, etc., decreased the contractile response of rat thoracic aorta to phenylephrine, increased the level of adiponectin, and enhanced the relaxation response of rat thoracic aorta to sodium nitroprusside	Wu et al. (2021b)
two hyperlipidemia models in zebrafish and VEC-VSMC co-culture	BBR(5、20 and 40 μM)	oxLDL-LOX-1-EMT-autophagy axis	By promoting autolysosome formation and degradation of LOX 1, inhibits vascular endothelial cells, and abnormal changes of vascular smooth muscle cells and vascular occlusion	Zheng et al. (2023)
Nitroglycerin-tolerant rat aorta	BBR(30 mg/kg/d)and the inhibitors of PKC and PKCα	PKC-α	reverses NTG tolerance through inhibiting PKCα activity in vascular smooth cells	Zhang et al. (2022)
Apoe−/− mice	BBR(1.0 g/kg/d)	Proliferator-activated receptor-γ and oxidative stress	BBR increased atherosclerotic plaque stability, increased acetylcholine-induced endothelium-dependent relaxation and superoxide dismutase activity, and decreased malondialdehyde content	Li et al. (2016a)
male C57BL/6J mice	Coptisine (0.1or 1 μM)	AMPK, eNOS and ER	Coptisine improves vascular function in diabetes through suppression of ER stress and oxidative stress	Zhou et al. (2021)
angiotensin IV-stimulated VSMCs	BBR(10、30 and 100 μmol/L)	PPARα-NO pathway in angiotensin IV-stimulated VSMCs	Berberine inhibited VSMCs proliferation induced by angiotensin IV in a concentration-dependent manner	Qiu et al. (2017)
VSMCs and artery of rat	Coptisine (6.71 μM and 5.62 μM)	Phosphorylation sites of Pak1S144/S203/Pak2S20/S197	CTS is a promising drug for the prevention of restenosis after angioplasty without adverse effects on reendothelialization	Chen et al. (2024)
apoE−/− mice	Coptisine (150 mg/kg/d)	MAPK signaling pathways and NF-κB nuclear translocation	CTS reduces atherosclerotic plaque area through its anti-inflammatory and lipid-lowering effects	Feng et al. (2017b)
porcine coronary artery rings	Berberidaceae (5.4%、15% and 1.6% BBR)	PDEs, especially PDE4 and PDE5	Active components of Berberidaceae induce endothelium-independent relaxation and act directly on VSMCs in coronary arteries to inhibit contractile responses	Alamgeer et al. (2016)
Calcified VSMCs	Quercetin (50 mM) and Berberine (25 mM)extracts	RUNX2 (a key marker of calcification phenotype), inflammatory factors and caspase-1	Anti-inflammatory and anti-vascular calcification	Ceccherini et al. (2024)

## 4 Clinical studies of BBR and its derivatives

MVEI is a precursor to cardiovascular diseases. In Asia, BBR is commonly used either as a standalone treatment or as an adjunct therapy for various conditions. The potential of BBR as a therapeutic agent for cardiovascular diseases has been widely demonstrated. In this section, we provide a concise overview of recent clinical studies on BBR, focusing primarily on its roles in regulating lipid metabolism, combating heart failure, modulating blood pressure, countering vasculitis, eliminating vascular toxins, and removing aberrant tissues.

It is well known that lipid metabolism disorders are a significant factor in myocardial endothelial cell injury. Therefore, enhancing lipid regulation is a crucial measure to slow the progression of cardiovascular diseases. A study involving 32 patients with hypercholesterolemia who took oral BBR preparations for 3 months (Kong et al., 2004) demonstrated that BBR intervention led to an average reduction of 29% in serum cholesterol, 35% in triglycerides, and 25% in LDL-cholesterol. This experiment showed even better results in hyperlipidemic hamsters.In a randomized, placebo-controlled, multicenter clinical trial involving 365 T2DM patients (Wang et al., 2022), the combined use of probiotics (Prob) and berberine (BBR) demonstrated superior lipid-lowering effects. Myocardial endothelial injury may increase the risk of heart failure (HF) by impairing blood flow supply and affecting the heart’s elasticity and adaptability. In patients with chronic heart failure (CHF), an 8-week follow-up after treatment with BBR at a dose of 1.2–2.0 g/day, in addition to standard medications, showed significant improvements in left ventricular ejection fraction (LVEF), exercise capacity, and clinical symptoms. Additionally, reductions in the frequency and complexity of ventricular premature contractions (VPCs) were observed compared to the control group (Zeng et al., 2003). This result highlights BBR’s potential in improving heart failure. The alterations in vascular shear stress caused by endothelial cell damage and the subsequent events of blood pressure dysregulation are worthy of further discussion.A comparative study involving 50 adult obese Mexican patients (Bandala et al., 2024) demonstrated that BBR exhibited significant clinical relevance in regulating blood pressure and the gene expression of metabolic risk-associated adipokines. Coronary arteritis, as a result of the accumulation of inflammatory factors and oxidative stress damage, is closely linked to myocardial endothelial cell injury.Xu’s study (Xu et al., 2020) demonstrated that BBR exerts a protective effect by inhibiting oxidative stress and endoplasmic reticulum stress in HCAECs (human coronary artery endothelial cells). In the mechanism section, we elaborate on the harmful effects of external stimuli such as viruses on myocardial vascular endothelial cells. BBR reduces viral replication in the vasculature and specifically affects the interaction between the virus and target organs (Warowicka et al., 2020).This result suggests the potential therapeutic efficacy of BBR in myocarditis. Myocardial vascular endothelial cell injury predisposes to large-scale vascular endothelial cell apoptosis, and aggressive measures in the early stages are critical, and BBR in combination with DDP may remove abnormal cells and tissues by inducing apoptosis and necrotic apoptosis (Liu et al., 2019).Additionally, there is a lack of direct studies on the efficacy of BBR in arrhythmias and coronary heart disease resulting from vascular endothelial cell damage. Future research is anticipated to address this gap and provide insights into the potential therapeutic benefits of BBR for these conditions.

## 5 Toxicity of BBR and its derivatives

Current studies have demonstrated that after absorption in the human body, BBR is primarily distributed in organs such as the liver, kidneys, spleen, lungs, and heart, and is mainly metabolized through the liver and kidneys. While the most common adverse reactions in clinical use include gastrointestinal reactions and allergic responses, accompanied by potential risks of liver and kidney damage, no high-quality, citable systematic reports or foundational experimental evidence have yet confirmed these findings (Imenshahidi et al., 2019). However, a few researchers have conducted relevant studies. Li YH. et al. (2016), by exploring the acute and subacute toxic reactions of BBR combined with 5-aminosalicylic acid (5-ASA) in the treatment of ulcerative colitis, found that when the two were used in combination, BBR inhibited lymphocyte viability and proliferation, leading to mild toxic effects on the spleen. Kheir et al. (2010) found that the administration route of BBR can affect the median lethal dose (LD50) in mice. Interestingly, although the bioavailability of different administration methods varies, the blood concentration of BBR that causes acute toxic reactions in mice remains constant and is independent of the administration route.Through a meta-analysis of 27 studies, Lan et al. (2015) found that the toxic side effects of BBR are closely related to the dosage. However, the frequency of these adverse reactions is very low, and the main side effects during treatment do not harm vital organs of the body.Additionally, a study by Xu et al. (2024). Showed that nanostructures assembled from BBR and magnolol not only effectively enhanced the oral bioavailability and biodistribution of BBR in the colon, improving therapeutic efficacy, but also demonstrated good safety, with no significant signs of toxicity observedTherefore, we conservatively consider BBR and its derivatives to be effective and low-toxicity traditional medicines.

## 6 Conclusions and future prospects

With the rapid advancements in pharmacology and global ethnomedicine, the exploration of active components in traditional medicines has deepened, yielding a series of exciting research findings that have significantly contributed to human health and wellbeing. BBR, a natural isoquinoline alkaloid, has demonstrated remarkable protective effects on myocardial vascular endothelial cells through its multifaceted mechanisms of action. These include inhibition of endoplasmic reticulum stress, anti-apoptotic effects, reduction of inflammatory responses, alleviation of oxidative stress, regulation of autophagy, suppression of platelet adhesion and aggregation, and modulation of gut microbiota metabolites. In recent years, research on BBR and its derivatives has significantly expanded, with notable progress achieved across multiple fields, including the circulatory, digestive, metabolic, and immune systems.

However, despite the significant progress, numerous unresolved challenges remain, signaling the immense value of further in-depth and extensive research in this field. Such efforts could open broader avenues for BBR applications in the future. Specifically, future research directions may focus on three key areas: first, further investigation of BBR’s mechanisms of action in complex and realistic pathological environments, such as myocardial vascular endothelial injury accompanied by dysregulated glucose and lipid metabolism or hemodynamic alterations. This would help fully unveil its potential for clinical practice. Second, addressing the challenge of BBR’s limited bioavailability by optimizing delivery strategies or combining it with other bioactive formulations could breathe new life into this traditional “old drug,” improving its pharmacodynamic and pharmacokinetic properties, enhancing therapeutic efficacy, and reducing potential adverse effects (Sultan et al., 2023; Zhang et al., 2017; Li et al., 2018). Finally, advanced research techniques and methodologies should be utilized to explore the controversial pharmacological properties of BBR and its derivatives, filling existing research gaps.

Although challenges remain, prior research has laid a solid theoretical foundation for BBR’s application in the field of myocardial vascular endothelial protection. It also provides robust support and guidance for future in-depth research and clinical applications.
